# Options for sampling and stratification for national forest inventories to implement REDD+ under the UNFCCC

**DOI:** 10.1186/1750-0680-5-9

**Published:** 2010-12-27

**Authors:** Danae Maniatis, Danilo Mollicone

**Affiliations:** 1 Environmental Change Institute, School of Geography and the Environment, South Parks Road, Oxford, OX1 3QY, UK; 2 Food and Agricultural Organisation of the United Nations, UN-REDD programme, Rome, Italy

## Abstract

**Background:**

Developing countries that are willing to participate in the recently adopted (16^th ^Session of the Conference of Parties (COP) in Cancun) mitigation mechanism of Reducing emissions from Deforestation and Forest Degradation - and the role of conservation, sustainable management of forests and enhancement of forest carbon stocks (REDD+) - will have to establish a national forest monitoring system in order to assess anthropogenic forest-related greenhouse gas emissions by sources and removals by sinks. Such a system should support the Measurement, Reporting and Verification (MRV) requirement of the United Nations Framework Convention on Climate Change (UNFCCC) as the REDD+ mechanism is results-based. A national forest inventory (NFI) is one potential key component of such an MRV system. Following the Decision adopted during the 15^th ^Session of the COP in Copenhagen, the most recent Intergovernmental Panel on Climate Change (IPCC) Guidance and Guidelines should be used as a basis for estimating anthropogenic forest-related greenhouse gas emissions by sources and removals by sinks and changes in forest carbon stocks and area.

**Results:**

First, we present the key indispensable elements of the IPCC Guidance and Guidelines that have been developed to fulfil the UNFCCC reporting requirements. This is done in order to set the framework to develop the MRV requirement in which a NFI for REDD+ implementation could be developed. Second, within this framework, we develop and propose a novel scheme for the stratification of forest land for REDD+. Finally, we present some non-exhaustive optional elements within this framework that a country could consider to successfully operationalise and implement its REDD+ NFI.

**Conclusion:**

Evidently, both the methodological guidance and political decisions on REDD+ under the UNFCCC will continue to evolve. Even so, and considering that there exists decades of experience in setting up traditional NFIs, developing a NFI that a country may use to directly support REDD+ activities under the UNFCCC represents the development of a new challenge in this field. It is therefore important that both the scientific community and national implementing agencies acquaint themselves with both the context and content of this challenge so that REDD+ mitigation actions may be implemented successfully and with environmental integrity. This paper provides important contributions to the subject through our proposal of the stratification of forest land for REDD+.

## Background

Since 2005 and in the context of the United Nations Framework Convention on Climate Change (UNFCCC) negotiations on a climate deal, activities related to forest land (FL) in developing countries have become one of the key possible mechanisms for climate change mitigation. In December 2009, the 15th meeting of the Conference of the Parties (COP) resulted in a Decision on '*Methodological guidance for activities relating to reducing emissions from deforestation and forest degradation and the role of conservation, sustainable management of forests and enhancement of forest carbon stocks in developing countries*', or REDD+ [1]. In December 2010, the 16th meeting of the COP resulted in a Decision including '*Policy approaches and positive incentives on issues relating to reducing emissions from deforestation and forest degradation in developing countries; and the role of conservation, sustainable management of forests and enhancement of forest carbon stocks in developing countries*'[2]. Countries willing to participate in this REDD+ mitigation mechanism under the UNFCCC will have to establish a national forest monitoring system ([1]Article 1(d)) that should support a Measurement, Reporting and Verification (MRV) requirement under the Convention. Furthermore, the Decision states that countries will have to use the Intergovernmental Panel on Climate Change's (IPCC) most recent Guidance and Guidelines as adopted or encouraged by the COP as a basis for estimating anthropogenic forest-related greenhouse gas (GHG) emissions by sources and removals by sinks, forest carbon stocks and forest area changes ([1] Article 1 (c)). In this way, emission estimates will be based on a common international methodological approach (IPCC) for MRV for REDD+. Suffice it to say that this is a new framework for forest science, research and projects to which both scientists and countries will have to adapt quickly.

Unfortunately and contrary to what might be expected, there appears to be a degree of disconnectedness between the 'academic' community working on REDD+ from a scientific perspective, and the understanding of this community of what the requirements are for REDD+ under the UNFCCC. One example is the many proposals to countries and papers in the literature (*e.g. *[3]) on the use of biomass mapping for REDD+ activities. However, none of these papers present a clear view on how to, for example, report on all the carbon pools and their fluxes or how to address the accuracy and uncertainty of biomass changes assessed over time. Subsequently they do not currently provide an indication on how countries could use a series of biomass maps to compile their GHG inventory for REDD+ related activities and report it to the UNFCCC Secretariat. Given the recent Decisions on REDD+, there is now an urgent need to assess and produce data on forest carbon stocks and forest carbon stock changes (Emission Factors or EF) on a country level with the direct objective of compiling a national GHG inventory and reporting it to the UNFCCC. In this new context to which countries will have to adjust to in order to participate in REDD+, it has become necessary to adapt 'traditional' forest inventories to forest inventories that can fully support REDD+ activities under the UNFCCC by following the IPCC Guidance [4] and Guidelines [5] combined with sound ecological and statistical strategies.

A National Forest Inventory (NFI) is one possible option for the Forest Monitoring system that REDD+ countries will have to establish in order to assess anthropogenic forest-related GHG emissions related to EF by sources and removals by sinks. Some REDD+ countries may have existing data and NFIs (*e.g. *Indonesia, Cameroon), others not or very old ones (*e.g. *the Democratic Republic of Congo). For countries that have existing data and NFIs on a national scale, the challenge will be to evaluate if and how those can be used to report under the UNFCCC. For countries that do not have NFIs, the challenge becomes to design and carry out a NFI in a relatively short time period (by 2012) with the specific objective of being able to report on GHGs following the IPCC Guidance [4] and Guidelines [5]. We propose that at least for FL, a NFI should provide the basis to estimate forest carbon stock changes and is the most comparable and accurate option to do so.

In the above context, our paper has two aims. First, we provide the UNFCCC and IPCC methodological framework in which a country will have to develop a NFI if it is to participate in the REDD+ mechanism. This is crucial as it lays out the basic framework for a NFI with that objective in mind. Second, the novel part of this paper provides several methodological options on how a country may manoeuvre to adapt its NFI to its national circumstances and capabilities for REDD+ implementation within this framework. Specifically, we propose a novel forest stratification system for a country's FL for REDD+ consistent with the IPCC Guidance [4] and Guidelines [5].

### REDD+ under the UNFCCC

**Figure 1:**
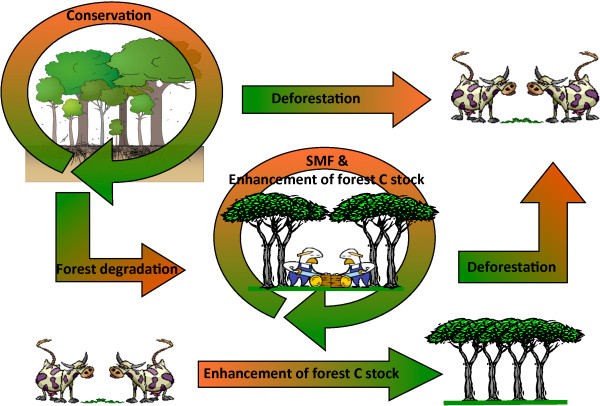
**REDD+ forest related activities**. In this figure arrows show the carbon budget behaviour of the potential activities. Arrows with a gradient from green to red represent potential source of greenhouse gases, while the arrow with a gradient from red to green represents a potential removal of greenhouse gases. Circular arrows represent a balance with possible positive (removal) and negative (source) results.

With regards to land-use, land-use change and forestry, five forest related activities have been identified in the REDD+ context [2]: (i) deforestation, (ii) forest degradation, (iii) conservation, (iv) sustainable management of forests and (v) enhancement of forest carbon stocks (Figure 1).

Using the IPCC methodological approach, REDD+ activities can be broken down into two main categories: first, land use change processes and second, change processes within the same land category.

Starting with the first, we can identify two forest related activities in the context of REDD+ that are land use change processes: (i) deforestation (*e.g. *from FL to other land use) and (ii) enhancement of forest carbon stock (*e.g. *from other land to FL). For these categories carbon stock change, supported by auxiliary data on land representation, could be reported using one NFI as a country may obtain country specific data on the different forest types but also on the EF for the other land use categories.

However, there are four forest related activities in the REDD+ context that are not land use change processes as they constitute FL remaining FL: (i) degradation (*e.g. *from unexploited to exploited forest or from unmanaged forest to managed forest); (ii) sustainable management of forest; (iii) conservation; and (iv) enhancement of forest carbon stocks. In this case, carbon stock and carbon stock changes could be reported using two NFIs or partially from one NFI. If one NFI is used, this NFI will have to provide accurate information on the carbon stock change dynamics.

## Results & Discussion

### The methodological framework to develop a NFI for REDD+

The COP requested the IPCC to develop methodological Guidance [4] and Guidelines [5] to assist countries in producing GHG inventories that are accurate in the sense of being neither over-, or underestimates and in which uncertainties are reduced as far as possible. In the framework of the UNFCCC's reporting requirements, it is important to understand the methodologies that were developed by the IPCC. These Guidance and Guidelines were not explicitly developed for REDD+, although this may be envisaged in the near future. By adapting the IPCC Guidelines, this section provides the methodological framework for a country choosing to participate in the REDD+ mechanism.

### The basic IPCC equation and its implications

In the IPCC Good Practice Guidance the most common simple methodological approach is to combine activity data (AD - information on the extent to which a human activity takes place) with emission factors (EF - coefficients which quantify the emissions or removals per unit activity):

(1) Emissions=AD×EF

Regarding the AD in general, the IPCC indicates that countries should accurately and completely represent and report all land areas in a country where human activities take place (land-use categories). This land representation should also reflect the historical trends in land-use area (20 years as a default value as suggested by the IPCC [4,5]) and information be reported to ensure transparency and comparability of estimates. Regarding EF, for REDD+ this will principally be represented by forest carbon stock changes.

### The IPCC's methodological approach for GHG inventories

Regarding the estimation of emissions and removals from FL, the IPCC has released extensive Guidance [4] and Guidelines [5]. Here we highlight some of the main points that need to be taken into account for REDD+.

#### The Tier levels and why they are important

Information on carbon stock changes can be obtained in various ways. The IPCC has categorized these approaches into three levels of increasing data requirements and analytical complexity called 'Tiers' [6]. Moving from Tier 1 to Tier 3 increases the accuracy (which is unknown for Tier 1) of the GHG estimates while increasing the complexity of the monitoring and analyses.

#### Key Categories - what are they?

**Figure 2:**
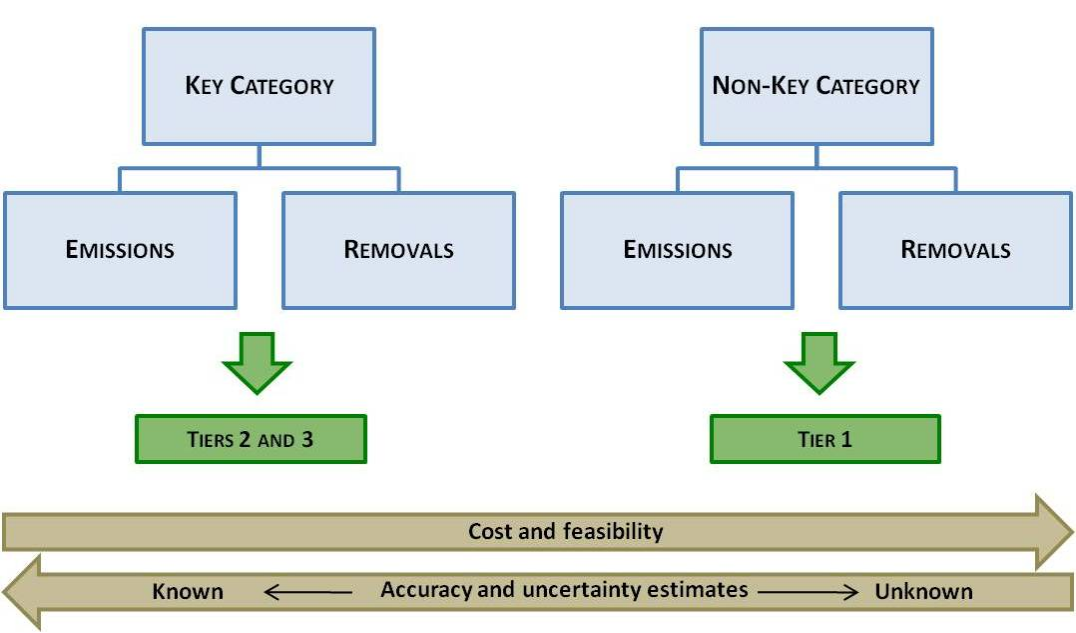
**Key Categories and Tier levels**. This figure illustrates the best practice in selecting Tiers between Key Categories and Non-Key Categories and provides an indication on the feasibility and the expected resulting accuracy and known uncertainty.

Several sources of emissions and removals by sinks exist on a land. These can vary considerably over time and depend on land-use and land-use changes. In the context of reducing GHG emissions and establishing GHG inventories, countries have to pay particular attention to their major sources of emissions as they are required to report on them with increasing accuracy. Large sources of emissions have been coined 'Key Categories' by the IPCC. Countries will therefore have to prioritise their resources and monitoring efforts to provide accurate estimates of such Key Categories. It is very likely that deforestation will represent a Key Category in many countries, for which it is good practice to use higher Tiers (2 or 3). However, national circumstances are always important and in the absence of better data Tier 1 could also be accepted for a Key Category in some cases. A representation of the interaction between the Key Categories and the Tier levels is illustrated in Figure 2. It is clear from this figure that as emissions and removals related to the five REDD+ activities will most likely be considered as a Key Category under the REDD+ mechanism, it is good practice that REDD+ countries use a Tier 2 level.

#### The different methods: Stock Difference or Gain-Loss Method?

The IPCC identifies two methods to assess carbon stock changes in the carbon pools: i) the process-based approach ('Gain-Loss Method'), which estimates the net balance of additions to and removals from a carbon stock, and ii) the stock-based approach ('Stock Difference Method'), which estimates the difference in carbon stocks at two points in time. The Gain-Loss method includes all processes that bring about changes in a pool including statistics on losses by harvest, fires, *etc*., while the Stock Difference Method measures the carbon stocks in relevant pools at two points in time to assess carbon stock change.

#### The 'managed land' proxy and land-use categories

A country will have to report on carbon stock changes (emissions and/or removals by sinks) only if these are human-induced. In this respect the IPCC advises the use of the 'managed land' concept as a proxy to discriminate human-induced emissions and removals. Only changes in managed land will have to be estimated and reported. If human activity occurs on land where there was previously no human activity ('unmanaged' land), it immediately becomes 'managed' land. In practical terms this means that a country territory will have to be divided into 'managed' and 'un-managed' land, or in other words, land where human activity occurs and land where human activity is absent. Countries will have to provide detailed definitions and the national approach to distinguish between unmanaged and managed will have to be described in a transparent manner [6]. Furthermore, a country will have to divide its national territory into the following six land-use categories that the IPCC has defined for GHG reporting [4]: (i) forest land; (ii) cropland; (iii) grassland; (iv) wetlands; (v) settlements and (vi) other land. When and where national land classifications systems are being developed for the first time, as will be the case for several REDD+ countries, it is good practice to ensure their compatibility with the six land-use classes described above [4]. These categories can be further subdivided into subdivisions which refer to national circumstances. When using a Tier 2 and 3 method, it is good practice to evaluate interactions between management practices that affect emission/stock change factors.

#### The five carbon pools that describe the carbon cycle and carbon fluxes

**Figure 3:**
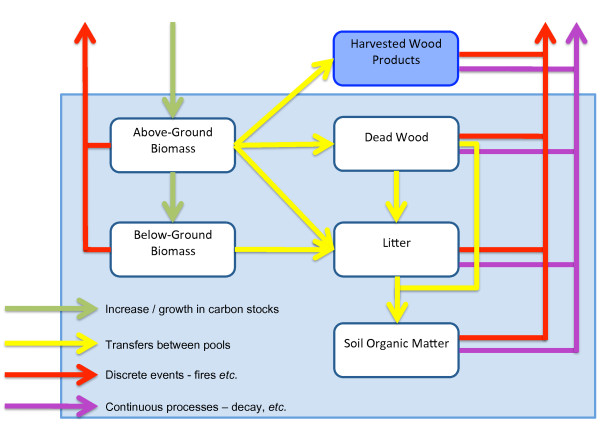
**Generalized carbon cycle of terrestrial AFOLU ecosystems**. This figure shows the flows of carbon into and out of the system as well as between the five carbon pools within the system (adapted from figure 2.1 [5]).

The IPCC defines five carbon pools: aboveground biomass, belowground biomass, dead wood, litter and soil organic matter which have to be measured and reported for GHG inventories. The generalised flowchart of the carbon cycle (Figure 3) shows all five pools and associated fluxes including inputs to and outputs from the system, as well as all possible transfers between the pools. The carbon cycle includes changes in carbon stocks due to both continuous processes (*i.e. *growth and decay) and discrete events (*i.e. *disturbances such as harvest, fire, insect outbreaks, land-use change and other events). Continuous processes can affect carbon stocks in all areas in each year, while discrete events cause emissions and redistribute ecosystem carbon in specific areas (*i.e. *where the disturbance occurs) in the year of the event.

#### Quality Control and Quality Assurance of the GHG inventory

It is important to assess the quality of measurements taken in the field, data compilation and data analysis in order to generate error estimates and improve future measurements. The IPCC 2006 Guidelines for National Greenhouse Gas Inventories [6] contain the necessary clarifications regarding Quality Control (QC) and Quality Assurance (QA) for GHG inventories. QC procedures are internal to the process of inventory preparation, while QA consist in an external (independent) assessment of the quality of the reported estimates. It should also be noted that, through its roster of experts, the UNFCCC Secretariat will verify the methods and the numbers in the national GHG Inventory that countries use to report.

### Minimum required elements (indispensable elements) for a GHG inventory

Following the methodological approach suggested by the IPCC, the minimum objective of countries that are willing to participate in a mitigation mechanism connected to a financial process (*e.g. *REDD+) under the UNFCCC, should be to compile a GHG inventory with estimates of carbon stock changes with a known uncertainty, applying Tier 2 or 3 for key changes. To meet this condition, a country needs to have: (i) country-specific estimates of EFs (by using at least a NFI for those associated to FL); (ii) multi-temporal inventory data and (iii) uncertainty estimates associated with any data reported.

The first methodological requirement to be met is country specific estimates of the EF. To obtain such estimates and to comply with the UNFCCC completeness reporting principle, it is primarily necessary to develop a REDD+ NFI measurement protocol that will provide estimates for the five IPCC carbon pools. The carbon stock change estimates that a country will have to submit through its GHG inventory will also have to consider all the possible transfers (yellow arrows) between pools (Figure 3).

The second requirement is the use of multi-temporal inventory data. Almost all the Annex I Parties that use a NFI to assess carbon stock changes for FL (39 Parties out of 41), use more than one NFI. The countries that are using data from only one NFI (*e.g. *Canada) are able to report on a temporal dynamic of the different carbon pools (in FL remaining FL) using models based on criteria such as the forest age class distribution. In the case of tropical countries, this solution could perhaps be adopted for some forest types, but in general this will not be practical for all the humid tropical forest types as forest stand structure is unevenly aged. Thus a different solution needs to be adopted for countries that would like to report on changes in carbon stocks for FL remaining FL through a single NFI. A possible approach could be through a stratification of FL based not only on the forest type, but also on its management practices and the REDD+ activities that countries will report on.

The final requirement is to provide uncertainty estimates with any data reported. This is an essential element of a complete NFI and for an inventory of GHG emissions and removals. They should be derived for both the national level and the trend estimate, as well as for the component parts such as EF, AD and other estimation parameters for each Key Category. Uncertainties should be reduced as far as is practicable during the process of compiling a NFI, and it is particularly important to ensure that any model used and the data collected are fair representations of the real forest status. Following the IPCC indication, quantitative uncertainty analysis should be performed by estimating the 95% confidence interval of the emissions and removals estimates for individual categories and for the total NFI. It is therefore crucial to develop a NFI sampling strategy where the probability of an element being included in an arbitrary sample of the population is known and where each element in the population has a positive inclusion probability.

### Methodological elements to optimize the REDD+ NFI according to national circumstances and capabilities

Unlike the indispensable elements (for the GHG inventory) listed above, some elements can be introduced into a REDD+ NFI methodology to make it more efficient or cost-effective, based on national circumstances and capabilities. In this section we will explore the elements of a NFI based on a multi-stage inventory. However, several other options exist to design a NFI, such as a multi-phase or unequal probability sampling system.

#### The context

**Figure 4:**
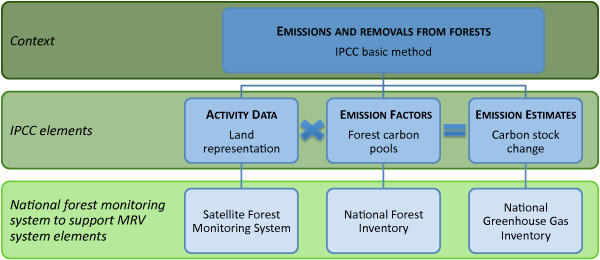
**A generalised MRV framework**. This figure illustrates the components and interactions of a national forest monitoring system to support MRV for the results-based REDD+ mechanism.

The first step is to clearly understand the components and interactions of a national forest monitoring system to support the MRV function of the results-based REDD+ mechanism, as presented in Figure 4. With regards to the EF component of the MRV system elements, the next important step is to stratify the FL for the NFI.

#### Stratifying the Forest Land

The stratification of land-use categories, especially of the FL in the different forest types, different forest management practices and REDD+ activities, is a key methodological challenge for countries for which, until presently, there has been no clear methodological proposal in the literature. Our objective in this section is to briefly explain some methodological reflections, combine them with the IPCC's methodological framework and propose a methodology to stratify the FL for REDD+ in order to report on carbon stock changes under the UNFCCC.

Terminology-wise, we use stratification as a quantitative criterion for classification, in other words, it helps a country to produce verifiable quantitative estimates for its forest strata. The stratification process will consist of separating the entire 'managed' FL in forest strata (relatively homogenous units) so that the variation within each forest type (stratum) is minimised at the expense of the variation between the forests (strata). Samples are subsequently taken from each forest stratum to obtain a more efficient estimate of the total population.

Besides the stratification of the forest types (which represent sub-land-use divisions), it is important to remember that REDD+ is an activity-based mechanism [2] (recall the five activities for REDD+). This could therefore result in five reporting tables for the GHG inventory to report on FL. It will thus be important that, from the outset, the stratification of the NFI will support the reporting for the five REDD+ activities (or the activities relevant to the country). For example, a country that selects the REDD+ activity of sustainable management of forest land could, for this activity, report only on forest area that falls under certified logging concessions (this could then represent a sub-stratum of the stratification).

A further important consideration, as outlined above, is that a country participating in the REDD+ mechanism will have to report on a carbon stock change dynamic. Preferably, this dynamic would reflect the different forest management practices. In many tropical countries in the last 20 years, the main source of emissions from FL remaining FL have originated from unmanaged and intact forest areas (un-exploited) that have been converted into managed forest areas (exploited) through selective logging or other degradation processes.

Ideally, one would use two or more NFIs to obtain multi-temporal inventory data and trends. In all Annex I Parties, this is the approach used. However, as stated previously, this is not the case for most non-Annex I Parties, of which most do not presently have a NFI or one targeted at providing this type of information. The challenge most non-Annex I Parties face is to design a replicable method for producing such multi-temporal inventory data while using one NFI which could reflect different forest management practices. This highlights the need for deeper stratification, for which we propose two proxies: first, the use of an intact forest landscape (IFL) and second, age class distribution.

Starting with the Intact Forest Landscape (IFL) proxy, this is defined as: *'...an unbroken expanse of natural ecosystems within the zone of current forest extent, showing no signs of significant human activity, and large enough that all native biodiversity, including viable populations of wide-ranging species, could be maintained' *[7]. To locate areas that satisfy the IFL definition, a set of criteria were developed by [7] and designed in such a way that they are globally applicable and easily replicable. Criteria were separated into two groups to be applied in sequence; the first group was used to assess the spatial extent of developed areas (*e.g. *distance from roads) and the second to assess fragmentation (*e.g. *minimum area extension). Such an approach allows for repeated assessments over time as well as verification by independent replication of assessments.

The second possible proxy is using an age class distribution. For forests with a clear age class distribution (for example plantation forests) an EF dynamic can easily be calculated. When trees have been removed due to anthropogenic disturbances or logging activities, it is fairly straightforward to estimate the initial forest carbon stock and forest carbon stock change due to the activity. For example, the degree of past disturbances can be estimated by calculating the coefficient of determination (R^2^) for a density-diameter relationship [8,9]. The value of R^2 ^indicates the extent to which a stand represents a balanced and evenly distributed structure, with a R^2 ^closer to one representing a more balanced structure [8]. Arranging such forest populations by age class and size class distribution can be used to quantify the magnitude of disturbances [10] or anthropogenic forest carbon stock changes. Unfortunately this proxy cannot be applied to most wet tropical forests, as rainforest species composition and structure is well known not to be in equilibrium [11]. This is reflected by an unequal and unclear age class distribution for species in most tropical forests. In tropical forests, trees of the same age may thus be large or small, depending on their individual growth history, making a species' size distribution an unreliable surrogate for its age distribution [12].

#### A new stratification approach

**Figure 5:**
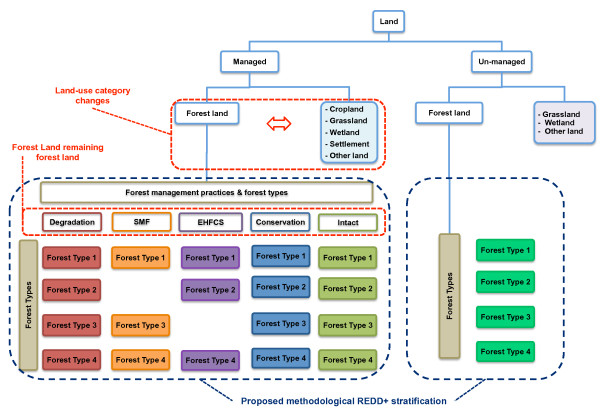
**Land stratification scheme based on forest type and forest management practices**. With this scheme a country may report on changes in carbon stock due to land use change activities (afforestation, reforestation and deforestation) reporting differences in carbon stock between forest land and cropland, grassland, wetland, settlement and other land. It may also report on changes in carbon stock in forest land remaining forest land while reporting on differences in carbon stock between and within the different forest management practices and forest types. SMF and EHFC stand for Sustainable Management of Forest and Enhancement of Forest Carbon Stocks respectively.

Considering all of the above, we propose that a country may adopt a land classification scheme such as the one presented in Figure 5. We propose the use of the IFL as a proxy to produce a carbon stock change estimate and to fulfil the requirement of generating multi-temporal inventory data while using one NFI only. This approach brings about two distinct/innovative advantages.

First, all forest types that fall under the 'managed' land will be further subdivided into a matrix of forest management practices (according to the REDD+ activities taking place on its FL remaining FL) and forest types.

Second, using the IFL, a forest management type of 'intact' will be incorporated into the matrix. For each forest type (where applicable) the 'intact' stratum will represent a stratum where no human activities are taking place. Although this would originally be defined as 'unmanaged' land (for which countries do not have to report), a buffer zone would be created in the 'unmanaged' land to be measured for each forest type where possible. In this way, for the same forest type which falls under a management activity of for example, forest degradation, the 'original' forest carbon stock of this forest type may be estimated using the 'intact' forest stratum.

Let us illustrate with two hypothetical examples. First, if the 'non-intact: degraded' Forest Type 1 (Figure 5) has an average forest carbon stock of 100 t C ha^-1 ^and the same Forest Type 1 in the management practice of 'intact' has a forest carbon stock of 150 t C ha^-1^, then the forest carbon stock change between the 'intact' state of Forest Type 1 and the forest management type of forest degradation of Forest Type 1 is -50 t C ha^-1^. Secondly, this stratification scheme can also be used to assess a forest carbon stock change within the strata by implementing suitable sampling strategies within the strata (*e.g. *sampling over chrono-sequence or sampling for age distribution, *etc*.). This approach of course only represents a proxy of a carbon stock change, but provides countries with an indication of a carbon stock change by using one NFI only.

This approach would allow countries to have separate sets of carbon stock change estimates per forest management activity and forest type for FL remaining FL. By using the 'intact' forest stratum as a proxy to measure the 'original' forest carbon stock, compared to the forest carbon stock where a given type of management practice is occurring, a country can estimate an approximate forest carbon stock change by using one NFI only. Countries could therefore report on changes in carbon stocks in FL remaining FL once they have multi-temporal AD on the extension (forest area) and changes in the extension (forest area change) of each of the forest types under each of the forest management practices. Let us illustrate with a hypothetical example once more. Let us say that that original forest area (AD) at time zero of our degraded Forest Type 1 with an original forest carbon stock of 100 t C ha^-1 ^is 150,000 ha. Let us now assume that at time zero plus two years, a country monitors that 50,000 ha (AD: forest area change) of our degraded Forest Type 1 is entirely deforested, let us assume for simplicity to 0 t C ha^-1 ^(note that this now constitutes a land-use change process as the FL is converted to another land type) and the rest of the forest area of degraded Forest Type 1 remains the same. By knowing the original forest area, the forest area change and the original forest carbon stock, a country may report on the forest carbon stock change (in this case emissions) resulting from activities on this area of land.

### Strategies to operationalise and successfully implement the REDD+ NFI

In order to operationalise the NFI successfully and effectively, we propose to carry out a NFI in several stages using some key traditional forestry elements adapted to reporting on carbon stock change for REDD+ under the Convention. While several other sampling design strategies exist, we will not discuss them here. The stages involved in generating a NFI vary considerably according to circumstances. However, in cases where there is no previous NFI or existing data that can be used, a NFI should attempt to ensure a learning-by-doing process where resources and efforts can be targeted simultaneously. If priorities change over time or resources become scarce, a country will be more able to respond adequately. In order to achieve this, we propose the generation of a NFI to be broken down into three overarching stages:

1. Forest area pre-assessment followed by the stratification of the FL;

2. Pre-sampling of the FL and;

3. Final sampling of the FL and assessment of carbon stocks and carbon stock changes.

We propose these three stages as guiding steps wherein the REDD+ NFI can be optimized according to national circumstances and capabilities. The optional elements are presented here under each of the three stages. However, if a country chose to adapt these three stages (*e.g. *into more or fewer stages), the optional elements can easily be shifted around.

### Stage I - Forest area pre-assessment

A country will need to obtain information on the spatial distribution of the land-use categories as defined by the IPCC and any sub-categories it chooses to use. For FL, it is essential to know the forest area extension, but also to have information on the spatial distribution of each forest type and the distribution of forest management practices and REDD+ activities that may affect carbon stock changes.

The IPCC proposes three different Approaches to measure AD. We present them here in order of increasing information content, but they are not hierarchical or mutually exclusive. Approach 1 identifies the total area for each individual land-use category, but does not provide information on changes of area between categories and is not spatially explicit. Approach 2 expands on Approach 1 by introducing tracking of land-use changes between categories. Approach 3 extends Approach 2 by tracking land-use changes on a spatial (*i.e. *geographically explicit) basis. Given all the methodological considerations, we believe that it is advisable for countries to use Approach 3. This implies the use of geographically explicit data which may be collected in the field or through remote sensing techniques. However, given that through the AD a country should assess forest area change on its territory, the tool to measure AD should also be a tool to observe trends in forest area change (*i.e. *annual or frequent AD estimates). To do so, an operational live satellite forest monitoring system is required (see Figure 4 on MRV scheme). The strategic methodological option of using remote sensing data rather than field data to assess AD simultaneously allows: (i) the assessment of forest area; (ii) the analysis of trends in forest area change (at present and retrospectively up to 20 years); and (iii) the significant reduction in the volume and cost of measurements needed to be undertaken in the field [13].

For countries where remote sensing data will have to be used for the AD, NFI field activities and measurements will contribute to the forest area assessment mainly as a training data set for remote sensing image analysis and as ground verification. The forest area pre-assessment is followed by the stratification of FL as described previously.

### Stage II - Pre-sampling

Before the pre-sampling stage effectively takes place on the field, the sampling strategy needs to be developed. Some components to take into account are: (i) sampling method; (ii) sample allocation; (iii) the positive inclusion probability of samples; (iv) sample distribution; (v) what constitutes a forest on the field; (vi) plot design; and (vii) field measurement protocols.

#### Sampling method

There are two basic ways to approach sampling. One is based on non-random methods where estimates can be provided for population parameters, but the accuracy of those estimates cannot be assessed. The second is based on methods of probability sampling. This approach also provides estimates for a population, but as it is based on laws of probability allows one to evaluate the uncertainty of the data [14]. The pre-sampling and final sampling schemes proposed are based on the probability sampling approach. However, for some issues like rare fire events or specific litter fall data collection for soil models, we suggest the use of non-random methods or unequal distribution sampling methods. For example, if a rare fire event results in forest carbon stock changes, it should still be measured by using a non-random method approach.

There are several ways to sample a particular land-use category such as forests. For the purposes of this paper we will focus on stratified random sampling (SRS). This method allows a country to have a stratification approach from the outset which could be similar to the one the country will have to use in its GHG inventory. SRS produces estimates that are unbiased provided that each stratum value is weighted according to the proportion the stratum forms of the entire population. The accuracy of the estimate can be assessed provided that a minimum of two sampling units occur within each stratum [14]. Stratification of the land-use categories, such as FL, entails the division of a sampling area into non-overlapping groups of strata, for example land cover and forest management practices. In order to calculate and optimise the number of 'samples' that need to be measured in each stratum for the inventory, it is necessary to test the heterogeneity of the variable to be measured (in this case AGB) in the strata. This information is needed so that a minimum amount of plots may be measured that are required to obtain a desired accuracy for the measured variable (AGB), which will be set by the REDD+ country.

We propose that the strata have an overlay of a systematic grid. Within each stratum a (or more) sample will be taken on this grid in a random way. This ensures that the variation between the sampling units in any one group (stratum) is less than the variation over the whole population. Several advantages exist to using this approach. First, it provides a separate estimate of the mean and the variance of the variable measured in each stratum (in this case AGB). Second, for a given sampling intensity, it yields more accurate estimates of the population parameters. Finally, it ensures better coverage of the population than simple random sampling [14]. The result is that there will be a different sampling density for the different forest types. This strategy will efficiently target resources and thereby make a REDD+ NFI as cost-efficient as possible.

#### Sample allocation

When using stratification, there are several ways to allocate samples to different strata (*e.g. *proportional allocation, optimum allocation and Neyman allocation [14]). In many cases, the boundary conditions for sample allocation will consist of budget constraints and accuracy requirements for the measured variable (in this case AGB) in each forest type. We propose the use of an *optimum allocation*. This method can be very powerful for countries as it is designed to give the most information per dollar spent - in other words, to cost the least for a given accuracy of the estimate (AGB) or, for a given cost, to produce a minimum variance of the estimate (AGB) [14]. Optimum allocation requires that estimates of both within-stratum variances and the costs of sampling are available. However, the optimal allocation with respect to different variables (*e.g. *number of trees, basal area, timber volume per species, total timber volume, *etc*.) are generally not equal. In the event that sampling has to provide information on various equally important parameters, a *compromise allocation *can be applied [15]. Depending on national circumstances and capabilities, the use of an optimal or compromise allocation is considered robust and cost-effective.

#### Sample distribution

**Figure 6:**
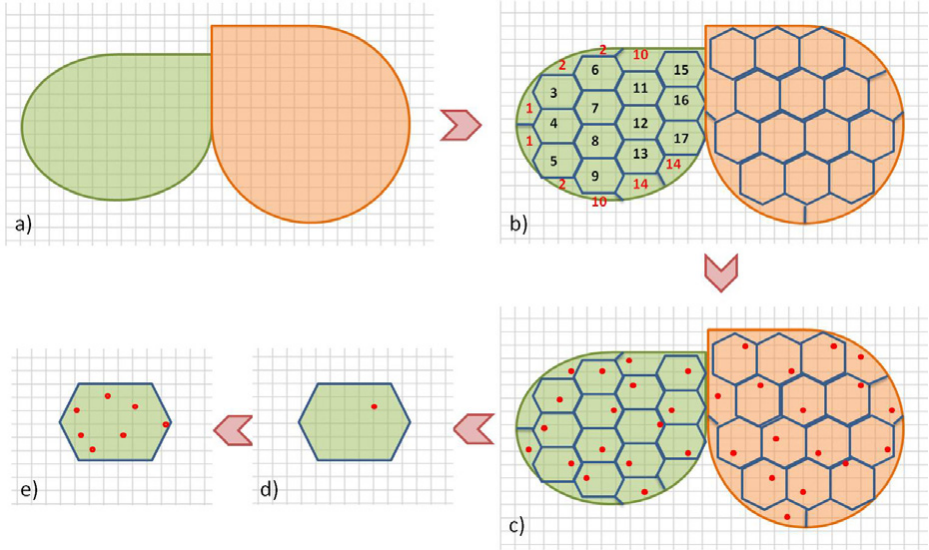
**Proposed sampling method, sample allocation and sample distribution for two hypothetical strata**. (a) two differest forest types overlayed with a systematic grid; (b) the same as (a) but with each forest type divided into the same number of area equivalent units (hexagons); (c) same as (b) but with one randomly selected sample point taken in each of the area equivalent units; (d) zoom in on one area equivalent unit and (e) the distrubution of seven sample points in the area equivalent unit using a distance rule for the sample location choice.

Random sampling with a randomised choice of sampling points can unfortunately lead to the selection of samples in which the spatial distribution is not optimal as points sampled in different strata can be close to each other, resulting in redundant information as spatial correlation occurs within and between strata. An option to overcome this problem is to divide each sub-land-use category, such as forest type, in an equal number of area equivalent units. We propose the division of each forest type (stratum) into 25 to 30 area equivalent units, where a measurement would be made in each unit (*e.g. *10 strata would equal 250 points). The choice of 25 to 30 area equivalent units and hence sample points is chosen as it is considered to be statistically sufficient for a pre-sampling exercise [16]. Sample points would be chosen at random in each area equivalent unit. However, in forest types with large surface areas, sample points may still be clustered within an area equivalent unit. A two-step procedure can be adopted to solve this problem: (i) the first sample point in each area equivalent unit is selected at random on the systematic grid; (ii) to avoid that the second sample within the area equivalent unit is too close to the first, a distance restriction can be imposed on the random sampling. This either forbids sample points below a certain distance or selects a replicate at the maximum possible distance of the first replicate. Additionally, some more plots could be selected to replace any of the selected plots that are impossible to measure. Figure 6 illustrates the sampling method, sample allocation and sample distribution for two hypothetical strata.

#### Positive inclusion probability of samples

To apply the probability sampling method, it is important that all the samples have a selection probability which is greater than zero. This is part of the 'indispensable elements' as it allows one to assess the accuracy of the results and to produce unbiased estimates of population totals. This could be achieved using a SRS with the sample distribution described above. It is preferable (in order to simplify the statistical analyses) that the samples within the 'managed' land have the same inclusion probability. However, regarding the 'unmanaged' lands which a country may choose to assess, there is a particular challenge of sampling in remote, inaccessible areas. Therefore, for the 'unmanaged' land, the positive inclusion probability could be lower as a country could choose to assess its unmanaged land based on a pre-defined limited budget.

#### To be a 'Forest' or not to be

A country participating in a REDD+ mechanism under the UNFCCC will have to submit a definition of its forests to the UNFCCC Secretariat. A country will have classified its land into predominant land use categories. The IPCC has provided specific methods to estimate emissions and removals for each of the land use categories. Moreover, the country may subdivide its land into managed and unmanaged to discriminate between natural and direct human-induced land processes (Figure 5). In the field, it is important to be able to define what constitutes FL or not according to a country's national definition and the IPCC land use categories. Although in theory the stratification based on remote sensing should result in plots to be measured falling in FL only, it is possible that the measurement point falls into a heterogeneous area on the field. This would result in the possibility that any land use category may be present at a given measurement point in the field. A method therefore needs to be developed to establish the dominant land use at the measurement point, as a country can only report on one land use category for each point. In heterogeneous areas where there is doubt as to whether a sampling point constitutes FL or not, a method should be developed to consistently, systematically and objectively assess this issue. Furthermore, a protocol should be set up on how to proceed if the randomly chosen plot does not appear to fit the 'forest' definition (such as a river, road, building, *etc*.) and as a consequence to measure another randomly selected point instead of the original plot. Another important reason for a rigorous approach to ensure whether or not sampling point constitutes a forest is that the NFI field activities and measurements should contribute to the forest area assessment (AD). This will improve the original remote sensing-based stratification by providing a field training data set for remote sensing image analysis and ground verification.

#### Plot design

As stated previously, in order to obtain carbon stock changes through an NFI the main variables of interest are forest carbon stocks and their dynamics, particularly in the aboveground biomass pool. At the measurement point, one could use different plot designs (different in size and shape) which would be adapted to the different strata. Plots are usually square, rectangular, circular or triangular and dimensionless (point sampling). It is possible to calculate unbiased estimates for all sample areas, regardless of their shape [17]. In tropical forests, access and visibility is often hindered by dense undergrowth. When vast areas have to be surveyed, it is common practice to use rectangular or square plots as such plots are easier to establish [17]. In plots where the undergrowth is less dense, circular plots could be employed as having the smallest periphery in relation to area and consequently the lowest number of borderline trees [17]. Generally speaking, there seems no reason to prefer one shape over another and there are difficulties involved with all shapes [18]. Nonetheless, the plot design (size and shape) should be designed to capture the real distribution of the population with the aim of producing unbiased and accurate biomass estimates. Plot size considerably influences AGB estimates [19] and for small plot sizes it was found that the distribution of AGB estimates was strongly skewed to the left of a normal distribution. This distribution became more symmetric as plot size increased. Furthermore, [20] reinforce previous findings advocating the use of a minimum plot size of 0.25 ha. Some strata may have higher within-stratum AGB variability and thus the plot design will need to take such variability into account and should avoid adding bias to the AGB distribution regardless of whether or not it is normally distributed. Any plot design should accommodate for reducing the error of including or not including a tree in the plot and the effects that slopes may have on AGB measurements. For example, [19] find that plots on slopes had significantly higher AGB estimates in the Barro Colorado Island in Panama. Therefore, tests for plot design should be carried out in each country for each stratum, resulting in a plot scheme being adopted for each stratum. The objective should be to adopt the plot design that for a given stratum is most suitable for AGB measurements while taking into account the above-mentioned points. Ultimately, plot design has to facilitate and optimize the number of samples that will have to be put in place for different degrees of accuracy (see equation 2 below).

#### Field measurement protocols

As stated before, a NFI for REDD+ will have to measure the five carbon pools as identified by the IPCC. Until now, we have mainly focused the discussion on the aboveground biomass component (pool 1). Several ways exist to measure the other carbon pools and we present some ideas which are non-exhaustive. Either way, each country will have to develop a full field manual including a field measurement protocol for each of the different forest types. The belowground biomass pool is very expensive to measure and allometric equations to calculate the belowground biomass as a proportion of the aboveground biomass could be used (*e.g. *[21]). Regarding the non-living biomass, the Center for Tropical Forest Science (CTFS) and the Amazon Forest Inventory Networks (RAINFOR) projects have recently developed specific protocols for tropical forests for the litter carbon pool [22] and the deadwood carbon pool [23], which could be used as a basis and be further adapted. The soil organic carbon pool can be inferred from soil profiles and/or measured directly or can be calculated using soil models. Coming back to the AGB pool, [24] suggest to measure Diameter at Breast Height (DBH), Height (H) and wood density. Wood density would be measured for certain tree species where little or no data is available in order to improve AGB estimates. Additionally, the sampling error within this measurement should be considered. This could be done using the method and guidelines already specifically developed for these purposes [24]. Information on wood density can also be collected using wood samples that are stored in 'xylaria' across the world (Maniatis *et al. *unpublished data).

### Stage III - Final sampling and assessment

In this final sampling stage, the first step for a country is to improve its stratification (forest type distribution, management practices distribution, *etc*.) based on the results of the pre-sampling. The overall approach of the NFI in this third stage will be to use a combination of temporary and permanent plots.

The second step is to calculate the population statistics. In order to calculate the number of 'plots' that need to be measured in each stratum for a REDD+ NFI, it is necessary to assess the statistics of the different forest types with particular reference to the variance of AGB in each forest type (stratum). This usually includes calculation and interpretation of the mean, variance and confidence limit of a population. This information is required so that for the REDD+ NFI, the minimum amount of plots may be undertaken required for an accuracy which the REDD+ country has set for a particular forest type (stratum) (*e.g. *95% with a 95% confidence interval (CI) for a dense forest - 80% with a 95% CI for a mangrove forest), and to assess the implementation costs. As a result of the pre-sampling stage, a country can calculate the required number of plots for each stratum for a given accuracy at a given CI (assuming that there is no systematic error in the estimates). The numerical formula is as follows [25]:

(2) $$n={\left(\frac{C\times t}{e}\right)}^{2}$$

Where *n *= the number of units required; *C *= coefficient of variation - a normalised measure of dispersion of a probability distribution and which is defined as the ratio of the standard deviation to the mean; *e *= required accuracy; *t *= student's t.

Similar to Stage II, there will be an optimal allocation of plots combined with a cost-effective and statistically sound solution to sample in the different forest types under different forest management practices and in the 'unmanaged'. It will be up to the country to decide if it wants to fully sample the 'unmanaged' forest area depending on its national resources and priorities.

For the final inventory, a QC and QA will have to be carried out. Based on the resources at its disposal, a country could envisage re-measuring a certain percentage of the sampled plots using the same methods but by an independent field team. Furthermore, the databases could be made publicly available (with different access levels) so that any party may check the structure of the database, calculations made and values reported. The QC and QA system are a priority to develop from the outset in a NFI context for REDD+.

A standard, uniform database should be designed for use by each country. This should be developed with the specific purposes of the NFI, in parallel with the development of the field sheets and with a view on the structure of the GHG inventory reporting tables. This would greatly facilitate data inputting and error checking and comparability among reported estimates.

### Integration of the REDD+ NFI with existing data and activities

In an ideal situation it would be preferable to incorporate existing data into the NFI. However, there are several points to keep in mind before this can be done. Firstly, a country will need access to the 'raw data' in order to assess the quality of the data.

Secondly, besides all the 'indispensable elements' that existing data will need to fulfil or provide the basis for, this data will need to include information on the five carbon pools. In the case of commercial forest inventory data, a problem is that many of them will only have collected data on the aboveground carbon pool and not the four other carbon pools (Maniatis *et al. *unpublished data). In the case of 'scientific' plots, the data might be more accessible and it is more likely that the five carbon pools will have been measured. On the other hand, scientific plots do not have the same wide distribution and representativeness as commercial forest inventory data and often suffer from the 'majestic forest' effect.

Nonetheless, depending on the national circumstances, it would be ideal to partially harmonise commercial forest inventory methodologies with the NFI as far as possible. In the future, sampling in logging concessions and projects (*e.g. *conservation initiatives) could be integrated with the NFI method that the REDD+ country would adopt. Alternatively, one could envisage that if a sample point falls within commercial logging concessions or within a research area, the executing agency of the REDD+ NFI in the country could request the company or institution in question to help with the measurement. Furthermore, one could envisage that commercial logging concessions with a certification for the sustainable management of the logged forests would represent the stratum of the REDD+ activity: sustainable management of forest. If the inventories would have a homogenous sampling method (or if the companies would agree to have one or have one imposed upon them by national legislation), they could be used and adapted to provide data on EF for the AGB pool and extended to provide information on the other carbon pools.

## Conclusion

We have presented the main elements of the IPCC Guidance and Guidelines that have been developed to fulfil the UNFCCC reporting requirements. A NFI that aims to support REDD+ activities should be designed in order to address these elements. Within this framework, we have developed and proposed a novel scheme for the FL stratification for REDD+. Furthermore, in order to successfully operationalise and implement their NFIs, we presented a strategic methodological option for the NFI (though this should not be considered as exhaustive) that countries may choose to consider based on their national circumstances and capabilities.

Decision on REDD+ now exist on both the methodological and political level [1,2]. Given the numerous challenges to the implementation of REDD+, the interdisciplinary nature of the issue and the shift in priorities from traditional NFI's, it is urgent for the scientific community (which will have to provide technical support, technology transfer and capacity building to REDD+ countries), to familiarise itself with the IPCC's Guidance and Guidelines, and consider methodological strategies to produce a NFI that may be used for REDD+ implementation and to report on emissions and removals from the FL in a GHG Inventory.

It is well understood that both the methodological guidance and political decisions on REDD+ under the UNFCCC will continue to evolve. Nonetheless, although the elements presented here will most likely have to evolve hand-in-hand with this process, they do provide the basic and secure framework for countries, scientists and various stakeholders to manoeuvre to ensure a country may successfully participate and report to the UNFCCC Secretariat data on its EFs under a REDD+ mechanism.

While the elements for the NFI method presented here are driven by carbon pools and especially the aboveground carbon pool, countries will be able to use this methodological framework to result in a multipurpose NFI, where biodiversity indicators, commercial volumes, *and so forth*, may be measured and incorporated. Additionally, such a NFI will be able to inform national policies and measures on FL.

Finally, our future work will be to combine and apply the theoretical and proposed conceptual strategy to develop and test methodological options for NFIs to support REDD+ implementation under the UNFCCC.

## Competing interests

The authors declare that they have no competing interests. The views expressed in this publication are those of the author(s) and do not necessarily reflect the views of the Food and Agriculture Organization of the United Nations.

## Authors' contributions

D Maniatis and D Mollicone conceived, drafted the manuscript and developed the methodological approaches. All authors read and approved the final manuscript.
